# TRAIL-*β* and TRAIL-*γ*: two novel splice variants of the human TNF-related apoptosis-inducing ligand (TRAIL) without apoptotic potential

**DOI:** 10.1038/sj.bjc.6600772

**Published:** 2003-03-18

**Authors:** A Krieg, T Krieg, M Wenzel, M Schmitt, U Ramp, B Fang, H E Gabbert, C D Gerharz, C Mahotka

**Affiliations:** 1 Institute of Pathology, Heinrich Heine-University, Moorenstr. 5, Duesseldorf D-40225, Germany; 2 Department of Gastroenterology, Hepatology and Infectious Diseases, Heinrich Heine-University, Moorenstr. 5, Duesseldorf D-40225, Germany; 3 Department of Thoracic and Cardiovascular Surgery, University of Texas, MD Anderson Cancer Center, Houston, TX, USA

**Keywords:** alternative splicing, apoptosis, neoplastic cells, TRAIL

## Abstract

Tumour necrosis factor (TNF) related apoptosis-inducing ligand (TRAIL/APO2L) is a recently identified member of the TNF family, which induces programmed cell death in a variety of neoplastic cell types, but not in most nonneoplastic cells. In this study, we report on the identification of two novel alternative splice variants of TRAIL in neoplastic and non-neoplastic human cells lacking either exon 3 (TRAIL-*β*) or exons 2 and 3 (TRAIL-*γ*). In both splice variants, loss of exon 3 resulted in a frame shift generating a stop codon with consecutive extensive truncation in the extracellular domain. Ectopic expression revealed a loss of proapoptotic potential for both alternative splice variants. In contrast to the predominantly cytoplasmatic localisation of GFP-tagged TRAIL-*α* and TRAIL-*β*, TRAIL-*γ* showed an additional association with the cell surface and nuclear membrane. In conclusion, alternative splicing might be involved in fine tuning of TRAIL-induced apoptosis and underlines the complexity of the TRAIL system.

TRAIL/APO2L (tumour necrosis factor related apoptosis-inducing ligand or TRAIL-*α*) is a recently identified type II transmembrane protein that belongs to the TNF family (Wiley *et al*, 1995; Pitti *et al*, 1996) and is processed proteolytically at the cell surface to form a soluble ligand (Mariani and Krammer, 1998). In contrast to other members of the TNF family, such as CD95L/FasL and TNF, TRAIL has been detected in a wide variety of both non-neoplastic and neoplastic cell types and tissues (Wiley *et al*, 1995; Pitti *et al*, 1996) and was found to induce programmed cell death in cancer cells, but not in most normal cells (Gura, 1997; Sheridan *et al*, 1997; Walczak *et al*, 1997; Ashkenazi *et al*, 1999; Dejosez *et al*, 2000). Consistently, TRAIL was shown to suppress growth of human tumours transplanted into mice (Walczak *et al*, 1999) and to improve survival of the tumour-bearing animals (Ashkenazi *et al*, 1999). Although repeated intravenous injections of TRAIL in primates did not cause detectable toxicity to non-neoplastic tissues (Ashkenazi *et al*, 1999), *in vitro* susceptibility to TRAIL-mediated apoptosis was reported recently for human hepatocytes (Jo *et al*, 2000).

Induction of apoptosis by TRAIL seems to be more complex than apoptosis mediated by other members of the TNF family, because four different TRAIL-binding receptors have been identified so far (Griffith and Lynch, 1998; Degli-Esposti, 1999). TRAIL-R1/DR4 and TRAIL-R2/DR5/KILLER/TRICK2 exhibit a death domain in their cytoplasmatic regions and signal apoptotic cell death upon overexpression (Gura, 1997; Pan *et al*, 1997a, 1997b; Sheridan *et al*, 1997). In contrast, ectopic overexpression of TRAIL-R3/DcR1/TRID, which lacks a cytoplasmatic region (Degli-Esposti *et al*, 1997b; MacFarlane *et al*, 1997; Pan *et al*, Q11997a; Sheridan *et al*, 1997; Mongkolsapaya *et al*, 1998), and TRAIL-R4/DcR2/TRUNDD, which exhibits only a truncated intracellular death domain (Degli-Esposti *et al*, 1997a; Marsters *et al*, Q11997; Kischkel *et al*, 2000), rather protects cells from TRAIL-induced apoptosis (Degli-Esposti *et al*, 1997a; Pan *et al*, 1997a; Kischkel *et al*, 2000). Although the molecular pathways of TRAIL-Q1induced programmed cell death are still under investigation, recent studies have demonstrated the recruitment of FADD and DAP3 as adaptors in the death-inducing signalling complex (DISC) and activation of caspase-8 (Bodmer *et al*, 2000a; Kischkel *et al*, 2000; Kuang *et al*, 2000; Sprick *et al*, 2000; Miyazaki and Reed, Q12001).

Several studies found a correlation between TRAIL resistance and expression levels of TRAIL in human cancer cell lines and keratinocytes (Rieger *et al*, 1999; Zhang *et al*, 1999; Dejosez *et al*, 2000). Moreover, the presence of intracellular caspase-8 (Eggert *et al*, 2001) and antiapoptotic proteins such as c-FLIP (Griffith *et al*, 1998; Leverkus *et al*, 2000) and/or activation of the transcription factor NF-*κ*B seem to determine TRAIL sensitivity (Jeremias *et al*, 1998; Goke *et al*, 2000; Keane *et al*, 2000; Bernard *et al*, 2001; Franco *et al*, 2001; Oya *et al*, 2001; Wuchter *et al*, 2001).

Transcriptional modification by alternative splicing is known to be involved in the regulation of apoptotic cell death in mammalian cells as well (Jiang and Wu, 1999). Thus, several apoptosis-related genes have been identified, for example, murine CD95L (Ayroldi *et al*, 1999), CD95 (Cascino *et al*, 1995), caspase-2, -8, -9 (Jiang *et al*, 1998; Srinivasula *et al*, 1999; Cote *et al*, 2001; Eckhart *et al*, 2001), cFLIP (Scaffidi *et al*, 1999) and survivin (Mahotka *et al*, 1999; Conway *et al*, 2000; Krieg *et al*, 2002), which are alternatively processed and thereby may exert distinct regulatory functions in the fine tuning of apoptosis.

In this report, we describe the identification of two novel TRAIL splice variants, i.e., TRAIL-*β* and TRAIL-*γ*, in non-neoplastic and neoplastic cells. The lack of exon 3 in TRAIL-*β* and of exons 2 and 3 in TRAIL-*γ* results in massive truncation of the extracellular binding domain and loss of proapoptotic potential.

## MATERIALS AND METHODS

### Cell lines and cultures

All RCC cell lines (*n*=30) used in this study were derived from typical representatives of the clear cell, chromophilic/papillary and chromophobe types of RCC, established in our laboratory as previously described (Gerharz *et al*, 1999). The cell lines were maintained with Dulbecco's modified Eagle's medium (DMEM, Gibco, Karlsruhe, Germany) supplemented with 10% fetal calf serum, penicillin and streptomycin. PBMCs were cultivated in RPMI Q2medium (Gibco, Karlsruhe, Germany) supplemented with 10% fetal calf serum, penicillin and streptomycin. All cell lines and PBMCs were cultured at 37°C in an atmosphere with 5% CO_2_.

### Stimulation of PBMCs

Buffy coats of blood samples from four healthy independent donors were collected according to standard procedures. For stimulation, cells were seeded at a density of 5 × 10^5^ cells ml^−1^ and treated with 10 *μ*g ml^−1^ concanavalin A (Sigma-Aldrich, Deisenhofen, Germany). Stimulated PBMCs were collected for RNA extraction after emergence of cell blasts (2–4 days).

### RNA extraction

Total RNA was isolated from the cultured RCC cell lines, PBMCs, mouse tissues and the mouse tumour cell line RAW 264.7 using the RNeasy kit (Qiagen, Hilden, Germany). The integrity of RNA (28S/18S ribosomal RNA) was controlled by electrophoresis followed by staining with ethidium bromide.

### Reverse transcription (RT) and PCR amplification of TRAIL

For cDNA synthesis, 2 *μ*g of total RNA was reversely transcribed in a final volume of 30 *μ*l containing 25 μM of each dNTP (Stratagene, Heidelberg, Germany), 100 pmol random hexamer primer (Stratagene), 20 U of recombinant RNasin RNase inhibitor (Promega, Heidelberg, Germany) as well as 5 U of AMV reverse transcriptase (Promega) with the corresponding RT buffer. The RT reactions were incubated at 55°C for 1 h.

PCR amplification of human TRAIL and GAPDH was performed in a final volume of 50 *μ*l containing 3 *μ*l first-strand cDNA solution, 2.5 U of *Taq* polymerase, 1 × PCR buffer, 25 *μ*M of each dNTP Q2(Qiagen, Hilden, Germany) and 25 pmol of each 3′ and 5′ TRAIL-specific oligonucleotide (forward primer, 5′-GAA TCC CAT GGC TAT GAT GGA GGT CCA G-3′ and reverse primer, 5′-GGA TTC GAG GAC CTC TTT CTC TCA CTA-3′) (GenBank accession number U37518) or 3′ and 5′ GAPDH-specific oligonucleotide (forward primer, 5′-ACG GAT TTG GTC GTA TTG GGC G-3′ and reverse primer, 5′-CTC CTG GAA GAT GGT GAT GG-3′) (GenBank accession number J04038). Conditions of PCR were as follows: initial denaturation step at 94°C for 2 min, followed by 35 cycles (TRAIL) or 27 cycles (GAPDH) of denaturation for 30 s, annealing for 1 min at 58°C (TRAIL) or 64°C (GAPDH), extension at 72°C for 1 min and a final extension step at 72°C for 5 min. PCR products were electrophoresed on 3% agarose gels containing ethidium bromide and visualised under UV transillumination.

Real-time PCR of murine TRAIL was performed on the LightCycler® (Roche Diagnostics, Mannheim, Germany). Amplification was performed in a total volume of 20 *μ*l in the presence of 2 *μ*l 10 × SYBR Green Fast Start Reaction Mix (Roche Diagnostics), 3 mM MgCl_2_, 25 pmol of each 3′ and 5′ TRAIL-specific oligonucleotide (forward primer, 5′-GAA TCC CTG CAT TGG GAA GTC AGA-3′ and reverse primer, 5′-GGA TCC TTA ATT AAA AAG GCT CCA AAG AAG-3′) (GenBank accession number U37522) and 2 *μ*l of cDNA (or water as negative control). Real-time PCR was carried out in glass capillaries with an initial denaturation step of 10 min at 95°C, followed by 65 cycles of 0 s at 95°C, 4 s annealing at 72°C, and elongation for 36 s at 72°C. Melting curve was directly performed after amplification with an initial denaturation of 0 s at 95°C, a temperature delay at 82°C for 30 s and a continous heating on 95°C for 0 s with a slope of 0.1°Cs^−1^. To confirm the results, PCR products were electrophoresed on 3% agarose gels containing ethidium bromide and visualised under UV transillumination.

### Sequence analysis

Bands of interest were excised from agarose gels and isolated using the QIAquick gel extraction kit (Qiagen), ligated into the pGEM-T-cloning vector (Promega) and cloned in accordance to standard protocols. Plasmid DNA was recovered employing the Plasmid Mini Kit (Qiagen), cycle sequenced using T7 or SP6 site-specific primers, and analysed with an ABI Prism 310 sequencing apparatus (Applied Biosystems, Weiterstadt, Germany).

### Cloning of TRAIL coding sequences

To generate GFP-tagged constructs of TRAIL variants, the coding cDNA sequences of the three TRAIL variants were amplified by PCR as described above. PCR amplification products were cloned into the pGEM-T-vector as described above, digested with *Eco*RI and *Bam*HI, ligated into the mammalian expression vector pEGFP-C1 (Clontech, Heidelberg, Germany) and cloned according to standard protocols. GFP-TRAIL-*α* construct was cloned as recently described (Kagawa *et al*, 2001).

### Transfection of cultured cells, assessment of cell viability and apoptosis as well as analysis of subcellular localisation by confocal laser scanning microscopy

Subconfluent TRAIL-sensitive HeLa cells were transiently transfected in 24-wells using polyfect transfection reagent, as described by the manufacturer (Qiagen). HeLa cells were incubated with the DNA and the transfection reagent for 24 h. To determine the number of cells that underwent apoptosis, HeLa cells were seeded on coverslips in 24-well plates, cultivated for 24 h and transfected as described above. After transfection, cells were washed twice with sterile PBS and fixed for 10 min in ice-cold methanol. Cells were treated with DAPI staining solution (0.1 *μ*g ml^−1^) for 5 min and washed twice with PBS. Up to 300 cells were counted in five different fields per preparation using an Axioscop fluorescence microscope (Zeiss, Germany). The percentage of apoptotic cells was calculated as the ratio between the number of GFP-positive cells with fragmented nuclei and all GFP-positive cells. The data presented are the mean ±s.d. from three replicates.

Cell number was analysed using the colorimetric MTT assay as described before (Mosman, 1983) and measured on a spectrophotometric Titertek Multiskan plate reader (Labsystems, Finland) at 570 nm. The percentage of viable cells in each well was calculated as the ratio between the absorbance of TRAIL-*α*-, TRAIL-*β*- or TRAIL-*γ*-transfected cells and the absorbance of vector control-transfected cells. The data presented are the mean ±s.d. from six replicates.

Statistical significance was considered for *P*-values of Student's *t*-test less than 0.05. Transfection efficiency was directly determined by the ratio of GFP-positive and -negative cells. The transfection efficiencies varied between 20 and 30% for HeLa cells.

To analyse the subcellular localisation of TRAIL-variants, HEK293 cells in the exponential growth phase were seeded onto sterile coverslips in six-well plates at a density of 1 × 10^5^ cells well^−1^. After 24 h, cells were transfected as described above. Afterwards, cells were washed two times in PBS, fixed in ice-cold methanol for 10 min and incubated with endoplasmatic reticulum-specific fluorescence dye concanavalin A Alexa-564 (Molecular Probes, Eugene, USA) (25 *μ*g ml^−1^) for 30 min. Coverslips were washed again in PBS, mounted and examined using a confocal laser scanning system TCS-NT (Leica, Bensheim, Germany).

### Western blot analysis

Protein extracts from transiently transfected HeLa cells were isolated by disrupting cells in lysis buffer (100 mM NaCl, 10 mM Tris-HCl pH 7.6, 1 mM EDTA pH8 and protease inhibitor). Protein samples (up to 50 *μ*g) were electrophoresed in 12% SDS–polyacrylamide gels at 70 mA for 4 h. Blotting to Opitran BA-S85 nitrocellulose membranes (Schleicher & Schuell, Dassel, Germany) was performed for up to 2 h at 650 mA in a tank of transfer buffer pH 8.3 (25 mM Tris-HCl, 1925 mM glycine, 20% methanol) using the Hoefer TE series Transphor electrophoresis unit (Hoefer Scientific Intsruments, San Fransisco, USA). To verify transfer efficiency and protein integrity, nitrocellulose membranes were stained with Ponceau S 0.2%. Afterwards, membranes were blocked in blocking buffer (100 mM Tris-HCl, pH 7.5, 150 mM NaCl, 0.2% Tween 20) plus 3% nonfat dry milk and 1% BSA for 2 h at room temperature. Immunodetection of GFP-TRAIL-fusion proteins was carried out by incubating membranes with a 1 : 2000 dilution of monoclonal mouse anti-GFP antibody JL-8 (Clontech, Palo Alto, USA) overnight at 4°C. PARP cleavage was detected by incubating membranes with a 1 : 500 dilution of monoclonal mouse anti- human PARP antibody, clone AC10.5 (Pharmingen, San Diego, USA) overnight at 4°C. Subsequently, membranes were incubated with a 1 : 1000 dilution with horseradish-peroxidase linked anti-mouse antibody (Amersham Pharmacia, Freiburg, Germany) for 1.5 h.

Proteins were detected by incubation with Lumi-Light substrate (Roche, Mannheim, Germany). Equal loading was confirmed by *β*-actin detection with a mouse anti-*β*-actin-specific antibody, clone AC-15 Q2(Sigma-Aldrich, Taufeirchren, Germany). Data on X-ray films were quantified by densitometry with the program Tina 20 (raytest Isotopenmessgeräte GmbH, Germany).

## RESULTS

### Identification of two novel TRAIL splice variants

**Figure 1:**
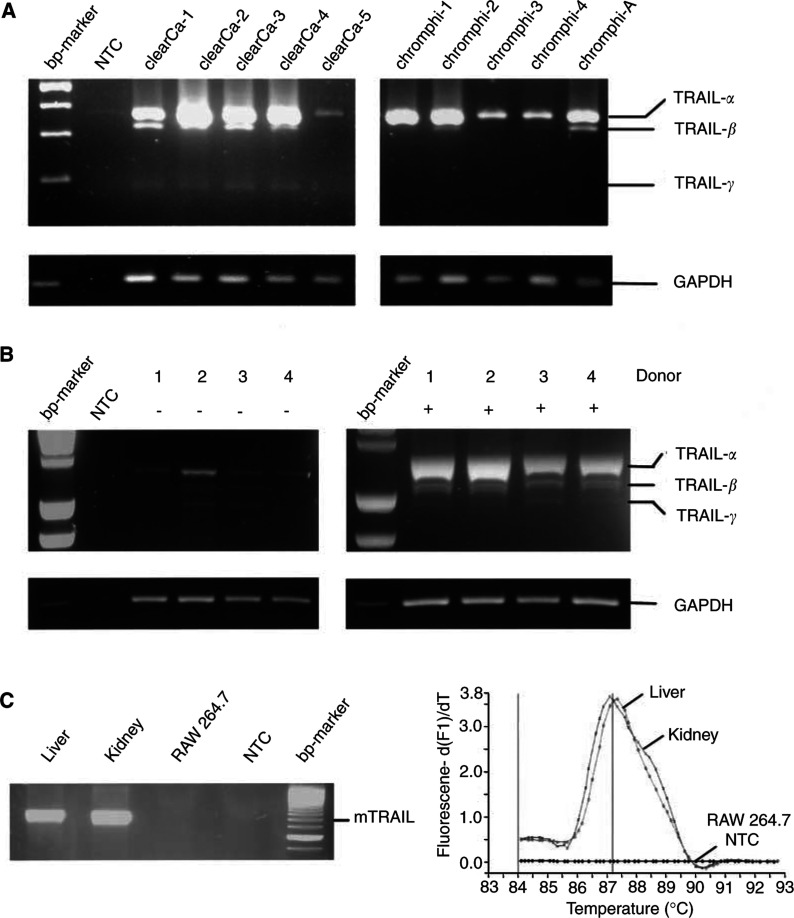
Expression of different TRAIL mRNA variants in neoplastic and non-neoplastic human cells. (**A**) TRAIL-*α* is the dominant transcript in renal cell carcinoma cell lines, whereas TRAIL-*β* and -*γ* show low expression levels only. Of note, expression of TRAIL-*γ* was not observed in chromophilic/papillary (chromphi-1 to -4) and chromophobe (chrompho-A) RCCs. (**B**) The alternative splice variants TRAIL-*β* and TRAIL-*γ* are expressed at low levels in both unstimulated (−) and concanavalin A-stimulated (+) PBMCs. (**C**) No alternative TRAIL splice variants could be detected in mouse liver and kidney or in the mouse tumour cell line RAW 264.7. Melting curve analysis showed a distinct peak for full-length TRAIL in liver and kidney tissue, but no additional signals for splice variants.

As revealed by RT–PCR analysis, all RCC cell lines (*n*=30) expressed TRAIL-*α*, irrespective of their histological types (Figure 1A

). Besides the expected amplification product of 371 bp, however, two additional bands of 328 and 190 bp were coamplified. These three amplification products were sequenced. The largest band was identified as regularly spliced TRAIL-*α*, whereas the 328 bp product was identified as a TRAIL variant with a deletion of 43 nucleotides corresponding to nucleotides 358–400 of the TRAIL-*α* cDNA of the human TRAIL sequence (GenBank Accession no. U37518). The smallest PCR product (190 bp) lacked nucleotides 220–357.

**Figure 2:**
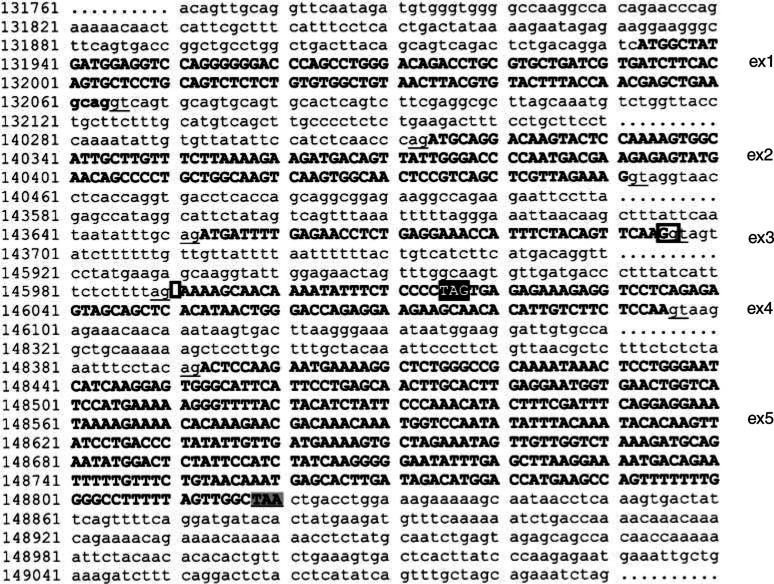
Genomic organisation of the human TRAIL gene. The cDNA sequence of human TRAIL (accession no. U37518) is marked in bold letters within the *Homo sapiens* chromosome 3, clone hRPK.44 A 1 (accession no. AC007051). SD and SA sites at the exon–intron boundaries are underlined. Of note, differences between our analysis of the genomic organisation and a recent publication (Gong and Almasan, 2000) are marked by squares. The stop codon generated by alternative splicing of exons 2 and 3 is marked by a black-shaded square.

Although the genomic organisation and the exon–intron boundaries of TRAIL had recently been published (Gong and Almasan, 2000), we repeated the database analysis by running advanced BLAST, looking for a genomic clone that contains parts of the human TRAIL gene. This database search revealed that the *Homo sapiens* Chromosome 3-clone hRPK.44 A (Accession no. AC007051) contained the entire coding sequence of TRAIL. Subsequently, we determined the exon–intron organisation of the human TRAIL gene, which consists of five exons (Figure 2

). Of note, we observed a difference in two nucleotides (position 143697 and 145990) marked by squares in Figure 2 when compared to the published sequence (Gong and Almasan, 2000). According to the genomic organisation, the 43 nucleotides deleted in the 328 bp long amplification product corresponded to exon 3 and, therefore, we designated this new alternative splice variant as TRAIL-*β*. The smaller band (190 bp) was found to lack both exon 2 (138 nucleotides) and exon 3 and, therefore, was designated as TRAIL-*γ*. Additional amplification of genomic DNA by PCR showed no product and excluded the presence of pseudogenes.

To investigate whether the novel TRAIL splice variants are exclusively generated in neoplastic cells, we performed RT–PCR with unstimulated and stimulated PBMCs showing that the alternative splice variants TRAIL-*β* and TRAIL-*γ* are expressed in non-neoplastic cells as well (Figure 1B). No alternative TRAIL splice variants, however, could be detected in mouse liver and kidney or in the mouse tumour cell line RAW 264.7 (Figure 1C). This observation strongly suggests that alternative splicing of TRAIL may be a species-restricted phenomenon.

### Splice donor and acceptor sites at the intron–exon and exon–intron boundaries of exons 2 and 3 are suitable for alternative splicing

**Figure 3:**
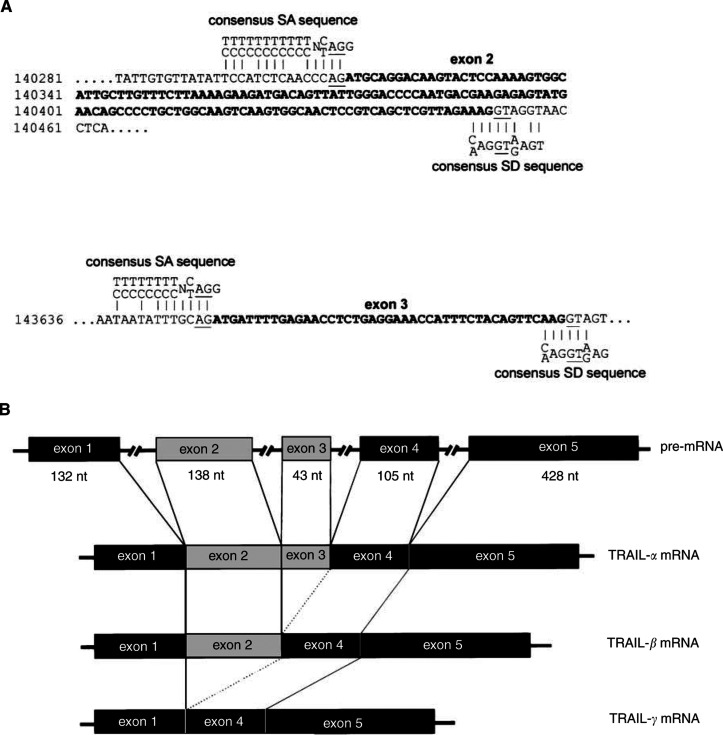
SD and SA sites flanking exons 2 and 3 (**A**) and the resulting TRAIL mRNAs (**B**).

Computational analysis with the Signal program (PC/GENE package) showed that exon 2 as well as exon 3 are flanked by splice donor (SD) and splice acceptor (SA) sites matching to the consensus sequence of common SD sites ({C/A}AG|GT{A/G}AGT) and SA sites ({T/C}_11_N{C/T}AG|G) as shown in Figure 3A

. Both the SA sites at position 140298–140457 of exon 2 and at position 143641–143700 of exon 3 contain the AG bases of SA sites as well as the GT bases of the SD sites that are necessary for post-transcriptional splicing on intron–exon boundaries. The necessary GT bases of SD sites for splicing processes exist also at the exon–intron boundaries of exons 2 and 3. Taken together, these data indicate that post-transcriptional processing of the TRAIL-pre-mRNA leads to the generation of three alternative splice products, i.e., TRAIL-*α*, TRAIL-*β* and TRAIL-*γ* (Figure 3B).

### Alterations of protein domains in the novel TRAIL isoforms and functional implications

**Figure 4:**
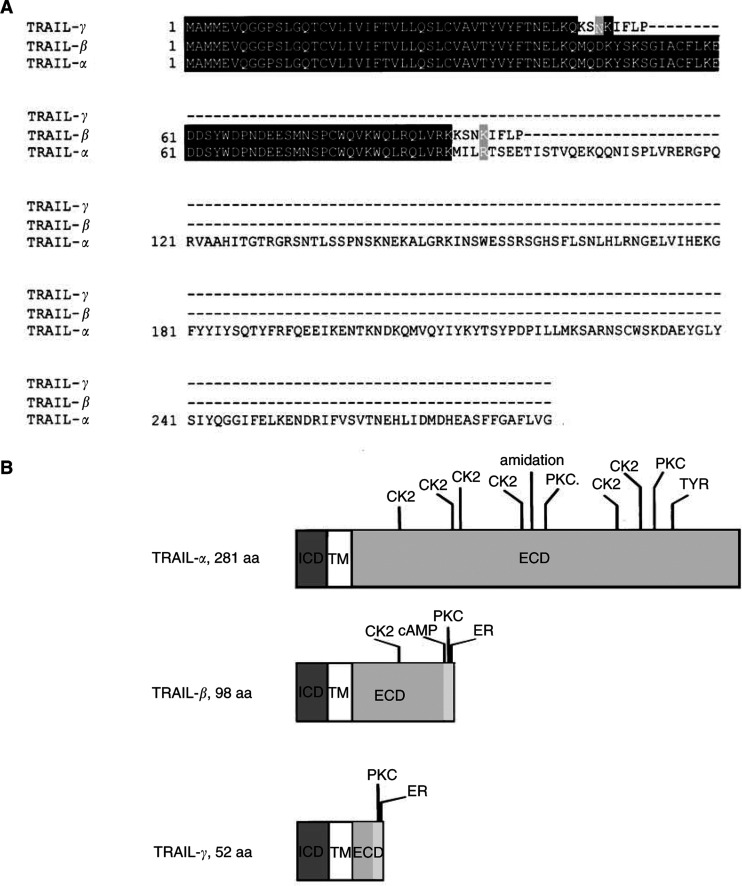
Structural alterations of protein domains in the novel TRAIL isoforms. (**A**) Multiple alignment of the three TRAIL isoforms was performed by Clustal W (Thompson *et al*, 1994) and enhanced with Boxshade. Dark shading: residues are highly conserved; light shading: residues are less well conserved; not shaded: residues are not conserved. (**B**) Protein sequences were analysed by PROSITE scan. Potential domains and modification sites are marked. A frame shift because of alternative splicing results in a potential endoplasmatic reticulum membrane retention (ER) signal at the C-terminus adjacent to new potential PKC-phoshorylation sites in both TRAIL-*β* and -*γ*. Light grey box = newly generated ER-signal containing domain. ICD = intracellular domain; ECD = extracellular domain; TM = transmembrane region; ER = endoplasmatic reticulum membrane retention signal; cAMP = cAMP-dependent protein kinase phoshorylation site; CK2 = casein kinase II phosphorylation site; TYR = tyrosine kinase phosphorylation site; PKC = protein kinase C phosphorylation site.

Computational analysis of TRAIL-*α* and its novel alternative splice variants showed that loss of exon 3 resulted in a frame shift generating a stop codon with consecutive truncation in the extracellular domains of both TRAIL-*β* and -*γ*. This truncation resulted in a 98 amino-acids long TRAIL-*β* and a 52 amino-acids long TRAIL-*γ* (Figure 4A

). Moreover, PSORT II analysis (Nakai and Horton, 1999) identified KIFL, a KKXX-like motif known as endoplasmatic reticulum membrane retention signal at the C-terminus of both TRAIL-*β* and -*γ*. PROSITE scan (Bairoch, 1991) also identified potential protein kinase C phosphorylation sites between amino acids 92–94 of TRAIL-*β* and amino acids 46–48 of TRAIL-*γ*, immediately adjacent to the potential endoplasmatic membrane retention signal. TRAIL-*β* also contains a potential cAMP- and cGMP-dependent protein kinase phosphorylation site between amino acids 89- and 92 (Figure 4B).

Although PROSITE scan analysis had identified a potential ER membrane retention signal at the C-terminus of both TRAIL-*β* and -*γ*, PSORT II analysis predicted a preferential cytoplasmatic localisation for all TRAIL isoforms (data not shown). To further investigate the subcellular localisation of TRAIL-*α* and its alternative splice variants, GFP-fused TRAIL variants were ectopically overexpressed in HEK293 cells. Using concanavalin A Alexa-594 as a marker of endoplasmatic reticulum, confocal laser microscopy did not reveal a selective accumulation of TRAIL-*β* or TRAIL-*γ* in the endoplasmatic reticulum (Figure 5

**Figure 5:**
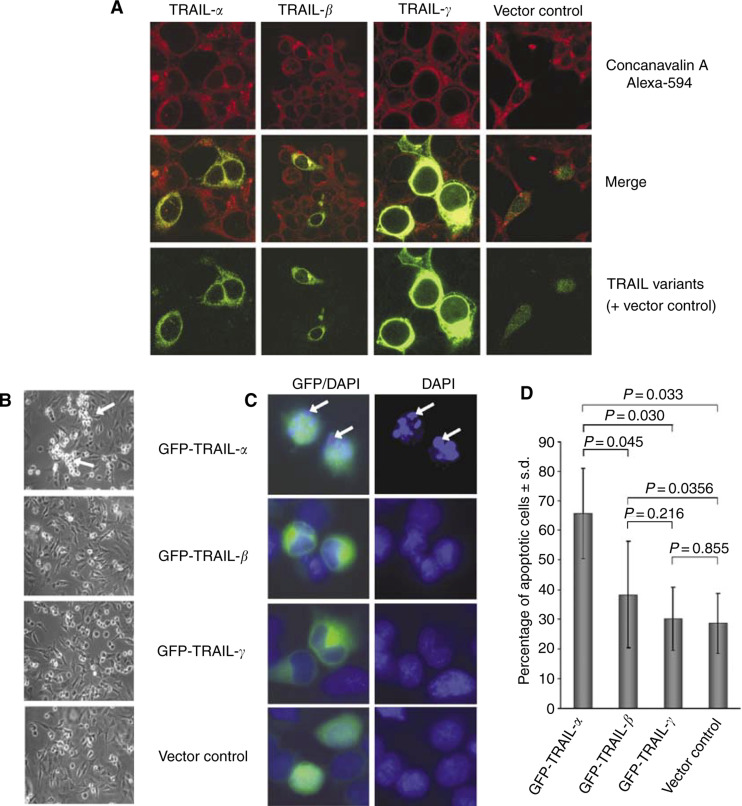
**A–D** Differential subcellular localisation and lost proapoptotic potential of the novel TRAIL variants. (**A**) Confocal laser scanning microscopy revealed a prominent cytoplasmatic localisation of all GFP-tagged TRAIL variants. The endoplasmatic reticulum was marked with the endoplasmatic reticulum-specific fluorescence dye concanavalin A Alexa-594. Yellow staining in ‘merge’ indicates colocalisation of all GFP-tagged TRAIL variants and the endoplasmatic reticulum. Of note, GFP-tagged TRAIL-*γ* additionally showed a prominent fluorescence of both the cell surface and the nuclear membrane, which was not observed for the other TRAIL variants. HeLa cells known to be sensitive to TRAIL-mediated apoptosis (Harper *et al*, 2001) were transfected with GFP constructs containing the different TRAIL isoforms or the empty vector. (**B**) Phase contrast microscopy of the transfected cells showed prominent clusters of apoptotic cells (arrows) in cultures of GFP- TRAIL-*α* transfected cells *but not* after transfection of other TRAIL variants or the vector control. (**C**) DAPI staining of nuclei permitted an identification of nuclei with fragmented or condensed chromatin, indicative for apoptotic cells (arrows), which could easily be distinguished from cells with intact nuclei under UV light. (**D**) Visual quantification of apoptosis was done by counting all GFP-positive cells as well as all GFP-positive cells with fragmented/condensed nuclei. The percentage of apoptotic cells was calculated as the ratio between the number of GFP-positive cells with fragmented or condensed nuclei and all GFP-positive cells. (Continued on next page.)

). However, TRAIL-*γ* was found to produce a more intensive fluorescence of the cell surface and nuclear membrane when compared to the other splice variants.

Using HeLa cells known to be sensitive to TRAIL-mediated apoptosis (Harper *et al*, 2001), ectopic expression of TRAIL variants revealed marked differences in the apoptotic activity of TRAIL-*α* and its novel alternative splice variants: ectopic expression of TRAIL-*α* led to huge clusters of apoptotic cells, which were not observed after transfection with the other TRAIL variants or the vector control (Figure 5B). DAPI staining permitted the identification of fragmented or condensed nuclei indicative for apoptosis (Figure 5C) and permitted a visual quantification of apoptosis (see Material and Methods). Using this assay, a significant decrease of apoptotic cells was observed after ectopic expression of GFP-TRAIL-*β* or GFP-TRAIL-*γ* or the vector control when compared to GFP-TRAIL-*α*-transfected HeLa cells (Figure 5D). The decrease of apoptotic potential observed for GFP-TRAIL-*β* and GFP-TRAIL-*γ* was paralleled by a significant increase of cell viability as demonstrated by MTT assays (Figure 5E

**Figure 5:**
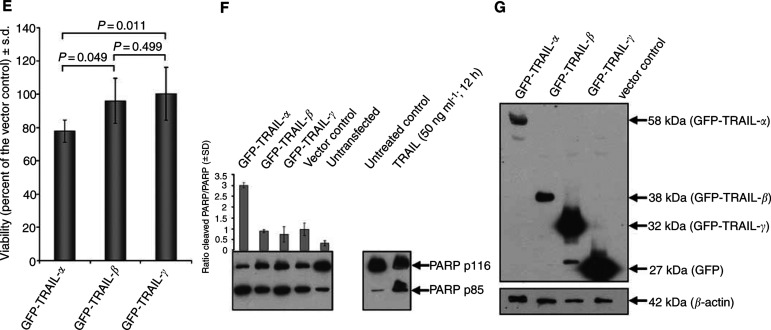
**Continued E–G** This visual apoptosis assay (*n* = 3) further confimed the loss of proapoptotic potential for GFP-TRAIL-*β* and GFP-TRAIL-*γ*, which also became evident (**E**) from an increase of surviving cells in our MTT assays (*n* = 6). (**F**) Immunodetection of the caspase-3-dependent ‘death substrate’ PARP showed enhancend cleavage only after ectopic expression of GFP-TRAIL-*α*. To ensure the detection of this cleavage product, protein lysates from TRAIL-treated control cells were separated on the same gel. The data presented are the mean ±s.d. from three independent transfections. (**G**) To show that the recombinant TRAIL variants are expressed on protein level, whole protein lysates of transfected HeLa cells were separated by SDS gels and immunodetection of GFP fusion proteins with anti-GFP-IgG was carried out after blotting on nitrocellulose membrane.

). To further confirm the effects of the different TRAIL variants on apoptosis, PARP cleavage was analysed by a monoclonal mouse antibody, that recognises a 85 kDa cleavage product besides the 116 kDa uncleaved PARP. PARP cleavage was found to be enhanced three-fold by ectopic expression of TRAIL-*α* in comparison with the other TRAIL variants or the vector control (Figure 5F).

To ensure that the observed effects were based on protein expression of all TRAIL variants, the expression of the fusion proteins was confirmed by Western blot analysis using a mononoclonal mouse anti-GFP anbtibody (Figure 5G).

## DISCUSSION

In this study, we report on the identification of two novel splice variants of TRAIL, i.e., TRAIL-*β* and TRAIL-*γ*, in RCC cell lines of all major histological subtypes. These previously unknown splice variants were not clearly detectable in RNase protection assays (Dejosez *et al*, 2000), because only 43 bp (TRAIL-*β*) or 181 bp (TRAIL-*γ*) differences in length were found in comparison with the primarily identified TRAIL-*α* transcript (Wiley *et al*, 1995; Pitti *et al*, 1996). It was especially intriguing to detect these splice variants in non-neoplastic human cells as well, whereas no corresponding splice variants were found in different cell types of murine origin.

Alternative splicing permits a high degree of protein diversity generating structurally and functionally distinct proteins that differ in their subcellular localisation, molecular targets or stability. Splice variants have previously been found to play a key role in the regulation of apoptosis as well, determining the actions of many apoptosis-related genes at all levels of apoptotic signalling pathways (Jiang and Wu, 1999). Thus, the CD95 Q3(APO-1, Fas) receptor exists in membrane-bound and soluble isoforms which antagonistically affect apoptosis (Jiang and Wu, 1999). The Bcl-2 family encompasses genes, for example, Bcl-X and its different splice variants Bcl-X_L_/X_S_, which antagonistically determine the susceptibility to cell death signals (Boise *et al*, 1993). Finally, different isoforms of caspases (Srinivasula *et al*, 1999; Cote *et al*, 2001; Eckhart *et al*, 2001) and inhibitor of apoptosis (IAP) proteins (Mahotka *et al*, 1999; Conway *et al*, 2000) are generated by alternative splicing. The novel TRAIL isoforms described in this study show an extensive loss of their extracellular binding domain, which plays a key role for trimeric stability, ligand–receptor binding capacity and apoptotic signalling (Pitti *et al*, 1996; Sheridan *et al*, 1997; Griffith and Lynch, 1998; Ashkenazi *et al*, 1999; Bodmer *et al*, 2000b; Kagawa *et al*, 2001). Consequently, the novel truncated TRAIL isoforms should be unable to form Q1stabile dimeric or trimeric receptor–ligand complexes and fail to trigger TRAIL mediated-apoptosis as demonstrated by our transfection experiments.

At first sight, therefore, TRAIL-*β* and TRAIL-*γ* may interfere with the fine tuning of TRAIL actions simply by reducing the amount of full-length ligand. In fact, the generation of truncated isoforms has been suggested as an additional mechanism to quantitatively regulate gene expression and action, because the mRNAs of all isoforms originate from the same pre-mRNA precursor pool (Jiang and Wu, 1999). Interestingly in this context, however, ectopically overexpressed TRAIL-*γ* exhibited a more prominent association with the cell surface and the nuclear membrane when compared to both TRAIL-*α* and TRAIL-*β*. Further experimental work, therefore, will have to show whether these alternative splice variants actually play a passive regulatory role for TRAIL-mediated apoptosis only or acquired novel functional properties as well. In any case, the identification of two novel splice variants may have implications for our understanding of TRAIL-mediated apoptosis in neoplastic and non-neoplastic human cells.
